# Feasibility and utility of MRI and dynamic ^18^F-FDG-PET in an orthotopic organoid-based patient-derived mouse model of endometrial cancer

**DOI:** 10.1186/s12967-021-03086-9

**Published:** 2021-09-26

**Authors:** Heidi Espedal, Hege F. Berg, Tina Fonnes, Kristine E. Fasmer, Camilla Krakstad, Ingfrid S. Haldorsen

**Affiliations:** 1 Department of Clinical Medicine, University of Bergen, 5021 Bergen, Norway; 2 Mohn Medical Imaging and Visualization Centre, Department of Radiology, Haukeland University Hospital, 5021 Bergen, Norway; 3 Centre for Cancer Biomarkers, Department of Clinical Science, University of Bergen, 5021 Bergen, Norway; 4 Department of Obstetrics and Gynecology, Haukeland University Hospital, 5021 Bergen, Norway

**Keywords:** Preclinical imaging, Endometrial cancer, Organoid-PDX models, Dynamic PET, Translational imaging, Multimodal imaging

## Abstract

**Background:**

Pelvic magnetic resonance imaging (MRI) and whole-body positron emission tomography-computed tomography (PET-CT) play an important role at primary diagnostic work-up and in detecting recurrent disease in endometrial cancer (EC) patients, however the preclinical use of these imaging methods is currently limited. We demonstrate the feasibility and utility of MRI and dynamic ^18^F-fluorodeoxyglucose (FDG)-PET imaging for monitoring tumor progression and assessing chemotherapy response in an orthotopic organoid-based patient-derived xenograft (O-PDX) mouse model of EC.

**Methods:**

18 O-PDX mice (grade 3 endometrioid EC, stage IIIC1), selectively underwent weekly T2-weighted MRI (total scans = 32), diffusion-weighted MRI (DWI) (total scans = 9) and dynamic ^18^F-FDG-PET (total scans = 26) during tumor progression. MRI tumor volumes (vMRI), tumor apparent diffusion coefficient values (ADC_mean_) and metabolic tumor parameters from ^18^F-FDG-PET including maximum and mean standard uptake values (SUV_max_/SUV_mean_), metabolic tumor volume (MTV), total lesion glycolysis (TLG) and metabolic rate of ^18^F-FDG (MR_FDG_) were calculated. Further, nine mice were included in a chemotherapy treatment study (treatment; n = 5, controls; n = 4) and tumor ADC_mean_-values were compared to changes in vMRI and cellular density from histology at endpoint. A Mann–Whitney test was used to evaluate differences between groups.

**Results:**

Tumors with large tumor volumes (vMRI) had higher metabolic activity (MTV and TLG) in a clear linear relationship (r^2^ = 0.92 and 0.89, respectively). Non-invasive calculation of MR_FDG_ from dynamic ^18^F-FDG-PET (mean MR_FDG_ = 0.39 μmol/min) was feasible using an image-derived input function. Treated mice had higher tumor ADC_mean_ (p = 0.03), lower vMRI (p = 0.03) and tumor cellular density (p = 0.02) than non-treated mice, all indicating treatment response.

**Conclusion:**

Preclinical imaging mirroring clinical imaging methods in EC is highly feasible for monitoring tumor progression and treatment response in the present orthotopic organoid mouse model.

**Supplementary Information:**

The online version contains supplementary material available at 10.1186/s12967-021-03086-9.

## Background

Successful translation of preclinical discoveries in oncology is unfortunately rare [1]. This may partly be due to lack of clinically relevant model systems and that preclinical imaging methods utilized for disease monitoring (e.g., optical imaging using fluorescence) are not feasible in the clinic [2]. Commonly used immortalized cancer cell lines are cost-effective and convenient to work with; however, they are genetically unstable and less representative of the clinical phenotype observed in patients [3]. Previous preclinical endometrial cancer (EC) studies have mostly relied on caliper size measurements of less relevant subcutaneous models using cell lines, or endpoint-only measurements in orthotopic models [4, 5]. Very few preclinical studies have used imaging methods mimicking those employed in the clinic [6]. We have recently developed EC organoid-based orthotopic mouse xenograft models (O-PDX) that recapitulate the histopathologic architecture, protein biomarker expression and the genetic profile of the donor tumor tissue [7]. These clinically relevant models respond well to conventional chemotherapeutic treatment and non-invasive imaging enables quantitative assessment of morphologic- and metabolic tumor characteristics indicative of tumor progression or treatment response [7, 8].

EC is the second most common gynecological cancer in industrialized countries and the incidence rate is increasing [9, 10]. Hysterectomy with bilateral removal of the ovaries is the primary treatment, which is curative in most patients with low-risk early-stage disease. However, about 15% of patients experience recurrence with a poor prognosis [11–13]. These patients, and patients presenting with advanced disease at time of diagnosis, usually receive adjuvant chemotherapy which is associated with toxicity and only moderate improvement of survival [14, 15]. Preoperative imaging plays an important role in risk stratification and surgical planning in EC. Local staging by pelvic MRI at primary diagnostic work-up is recommended for preoperative assessment of deep myometrial invasion, cervical stroma invasion, extrauterine tumor extension and metastatic pelvic lymph nodes [16]. MRI is also the preferred modality to assess local recurrence. Diffusion-weighted MRI (DWI) depicts the microscopic mobility of water in the tissue, which is strongly influenced by tissue microstructure, microcirculation and cellularity. DWI allows calculation of apparent diffusion coefficient (ADC)-maps, and low tumor ADC is linked to high tumor cellularity and predicts aggressive EC disease [17]. Preoperative, static ^18^F-fluorodeoxyglucose (^18^F-FDG)-PET-CT is often recommended in putative high-risk disease and in patients with clinical suspicion of recurrent disease, as ^18^F-FDG-PET-CT yields high accuracy for detecting lymph node metastases and distant spread in EC [18, 19]. Primary ECs are typically highly ^18^F-FDG avid [18]. Interestingly, a recent study using a dynamic ^18^F-FDG-PET-CT protocol demonstrated the clinical feasibility and superior performance of the dynamic imaging parameter, metabolic rate of ^18^F-FDG (MR_FDG_), compared to the static imaging parameters; i.e., standardized uptake values (SUV) in 101 patients diagnosed with cancers from different origins [20].

Preclinical use of standard clinical imaging methods in endometrial cancer (EC) is currently limited. We demonstrate the feasibility and utility of MRI and dynamic ^18^F-FDG-PET imaging for monitoring tumor progression and assessing chemotherapy response in an orthotopic O-PDX mouse model of EC.

## Methods

### Animal model

**Fig. 1:**
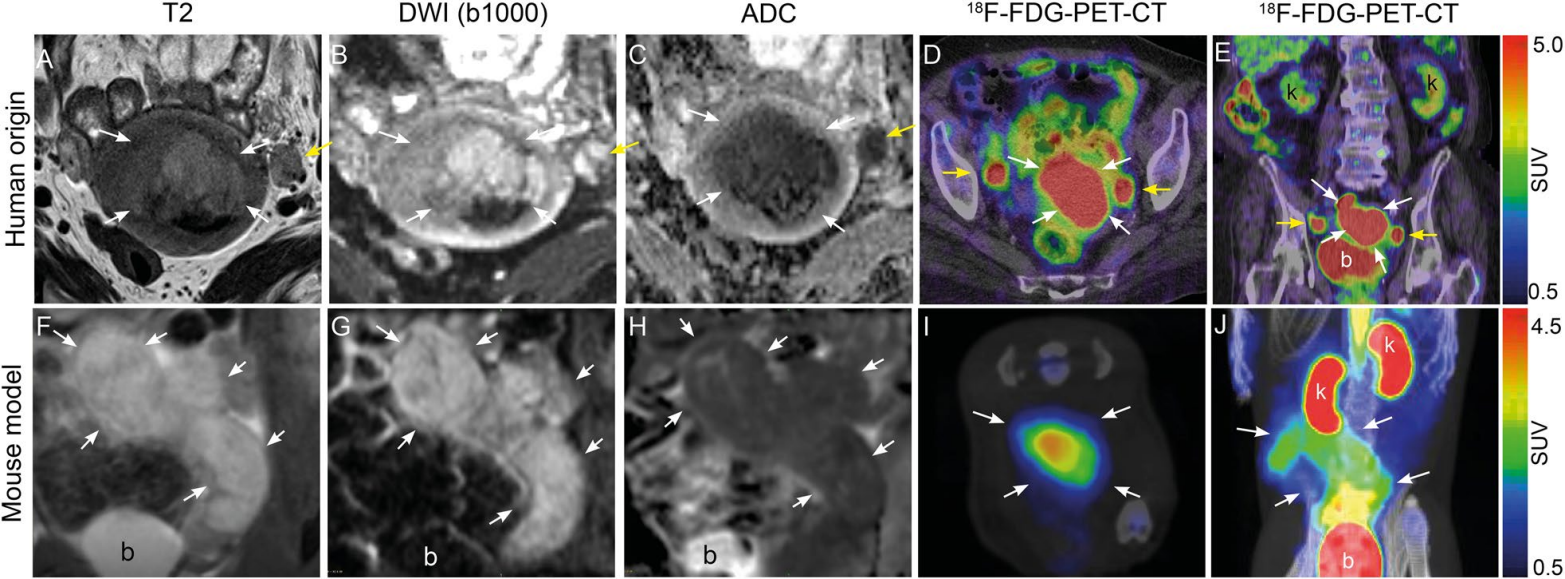
Preoperative MRI and ^18^F-FDG-PET imaging in the donor patient and corresponding preclinical MRI and ^18^F-FDG-PET imaging in the developed orthotopic O-PDX mouse model. Upper panel: Axial-oblique MRI sequences (**A**–**C**) displaying an irregularly shaped large uterine primary tumor invading > 50% the myometrial wall (white arrows) and enlarged pelvic left sided lymph node (yellow arrows), all exhibiting restricted diffusion (**B** and **C**). On PET-CT high ^18^F-FDG avidity is seen both in the primary tumor (white arrows) and in the bilateral pelvic lymph nodes (yellow arrows) (axial (**D**) and coronal (**E**) planes). Lower panel: Coronal MRI of a representative mouse tumor (white arrows) in the left uterine horn (**F**–**H**) 3 weeks after implantation displaying characteristic hyperintensity on T2 (**F**) and high b-value image (DWI) (**G**) and corresponding hypointense on the ADC map (**H**) indicating restricted diffusion. Abdominal axial (**I**) and maximum intensity projection (MIP) images from ^18^F-FDG-PET-CT (**J**) depict a highly ^18^F-FDG- avid uterine tumor in the same mouse, 2 days after the MRI examination. *b* bladder, *k* kidney, *ADC* apparent diffusion coefficient, *DWI* diffusion-weighted MRI, ^*18*^*F-FDG* fluorodeoxyglucose, *O-PDX* organoid-based patient-derived xenograft, *SUV* standardized uptake value

Hysterectomy specimen was donated by a consenting woman (approval ID 2015/2333 and 2018/548 REK vest) diagnosed with grade 3, endometrioid EC, and International Federation of Gynecology and Obstetrics (FIGO) stage IIIC1. Preoperative pelvic MRI and ^18^F-FDG-PET in this patient (Fig. 1A–E) was acquired as part of the routine diagnostic work-up. Organoids were established, expanded and orthotopically implanted as described previously [7, 21]. Briefly, fresh tumor tissue was enzymatically dissociated before embedding into growth factor reduced (GFR) Matrigel (Corning) (1:2). Organoid:Matrigel suspension was seeded as 25 µl droplets in 48-well plates and covered with expansion medium. Organoid expansion was performed weekly by breaking the organoids mechanically into smaller fragments, followed by resuspension in fresh GFR Matrigel and seeding. At passage 14, organoids were immersed 1:1 in GFR Matrigel prior to orthotopic implantation (2 × 10^6^ cells) into the left uterine horn of female NOD/SCID IL2rγ^null^ (NSG) mice (21 mice in total). All animal experiments were conducted in accordance with Norwegian and European regulations (approval ID 20194). Mice were monitored for disease symptoms including lethargy, ataxia and weight loss (> 10%) and were sacrificed following any of these symptoms or at the end of the study (8 weeks post-implantation).

### Study design

18 mice were imaged by weekly MRI and PET-scanning from Week 3–5 post-implantation in order to monitor primary tumor growth. Table 1 includes a detailed overview of all imaging sequences employed for each mouse in the different weeks. The PET images were acquired two days post-MRI due the scanners being located in different buildings; this setup allowed one day acclimatization after transport. Correlation analyses for the MRI- and PET imaging parameters included examinations acquired within 3 days. A subcohort of nine mice were further included in a treatment study with imaging after chemotherapy (Table 1). These were randomized into treatment- (n = 5) or control groups (n = 4) and received combined carboplatin (15 mg/kg)/paclitaxel (12 mg/kg) (treatment group) or saline (100 μl, control group) intraperitoneally (ip) twice per week, starting at vMRI > 145 mm^3^ and continuing until the end of study. Imaging included T2-weighted MRI and DWI prior to sacrifice for all. Additionally, one mouse from each group was followed longitudinally with weekly T2-weighted MRI and DWI during Weeks 4–8. For DWI analyses, the 4 control mice in the treatment study were combined with the mice scanned outside the treatment study, in order to capture a larger variety of tumor sizes and increase the statistical power (Table 1).

**Table 1:** Overview of imaging examinations in naïve mice and treatment study

Mouse	Week 3	Week 4	Week 5	Total scans, week 3–5	Treatment study (end-point imaging, week 7/8) DWI + T2
	Naïve, untreated tumors				
M1	T2 + DWI + PET				
M2	T2 + DWI + PET	T2 + PET	T2		Control (L)
M3	T2 + DWI + PET		T2 + PET		
M4	T2 + DWI				
M5	T2 + DWI	T2 + PET			
M6	T2 + PET	T2 + PET	T2 + PET		
M7	T2 + PET	T2 + PET	T2 + PET		
M8	T2 + PET	T2 + PET	T2		
M9	T2 + PET	T2 + PET^a^	T2		
M10	T2 + PET	T2 + PET^a^	T2		Control
M11	T2 + PET				Treatment (L)
M12	T2 + PET				
M13	T2 + PET				Treatment
M14	T2 + PET				
M15		T2 + PET			
M16		T2 + PET			
M17		T2 + PET			Treatment
M18			T2 + PET		Control
M19					Control
M20					Treatment
M21					Treatment
T2—scans	14	10	8	**32**	9
DWI—scans	5	–	–	**5**	9
PET^b^—scans	12	10	4	**26**	0

L indicates weekly longitudinal T2 + DWI imaging (in two mice) in the treatment study from week 4 until week 7/8 (control/treated mouse) *DWI* diffusion-weighted MRI, *L* longitudinal, *T2* T2-weighted MRI

^a^Static PET only due to technical issues

^b^PET refers to PET-CT imaging, however CT images were used as PET anatomical reference and attenuation correction only

### MRI scanning and image reconstruction

Images were acquired on a small-animal 7 Tesla MRI scanner (Pharmascan, Bruker) using a mouse body quadrature volume resonator in a single-coil configuration. Mice were anesthetized by sevoflurane mixed in oxygen and breathing and body temperature were monitored during scanning. T2-weighted sequences were acquired coronally (TE/TR 25/2500 ms, 5 averages, matrix 160 × 160, field of view 32 × 32 mm, slice thickness 1 mm, resolution 0.2 × 0.2 mm) and included the whole tumor volume. Coronal DW-images (TE/TR 17/3000 ms, 3 averages, matrix 67 × 93, field of view 20 × 28 mm, slice thickness 1 mm, resolution 0.3 × 0.3 mm) were generated using b-values of 0 and 1000 s/mm^2^. ADC parametric maps were automatically generated from the DWI-series using the manufacturer’s software (Paravision 6.0).

### MR image analyses

Manual segmentation aiming at including all primary tumor tissue on the coronal T2-weighted images were performed using the free software ITK-SNAP (Version 3.8) [22]. The anatomic tumor volume (vMRI) was calculated by summing the segmented volumes from all slices depicting tumor tissue. One reader with 9 years of preclinical MRI experience (H. Espedal) performed the segmentation with case-by-case consensus discussion with another observer (clinical radiologist I.S Haldorsen) with 6/12 years of gynecological cancer imaging experience (preclinical/clinical). Segmentation was carried out blinded to treatment group. The average tumor ADC (ADC_mean_) was similarly measured by segmenting tumor tissue in all slices on the ADC-maps using ITK-SNAP. The reported ADC_mean_ represents the mean value throughout the whole tumor.

### PET-CT scanning and image reconstruction

The PET images were acquired on a small-animal PET-CT scanner (Nanoscan, Mediso) and mice were scanned in pairs using a dual bed. Prior to imaging, mice were fasted (average 19 ± 2 h) to minimize gastrointestinal background uptake. Mice were anesthetized using sevoflurane mixed in oxygen, and ^18^F-FDG was diluted in saline to a total volume of 150 μl at average injected dose 8.3 ± 1.2 MBq. ^18^F-FDG was injected in the lateral tail vein at start of the 1-h dynamic PET acquisition. Two mice were imaged with a static protocol only (30 min uptake time followed by 30 min acquisition), due to technical issues. Prior to the PET, a low-dose CT (50 kVp, 0.2 mAs, 0.38 mm slice thickness) was acquired for anatomical reference and attenuation correction. The mice were monitored for breathing and temperature during scanning. Static images were reconstructed using the list-mode data from 30 to 60 min post ^18^F-FDG injection. Dynamic images were reconstructed into the following time frames: 5 × 2 s, 5 × 10 s, 2 × 120 s, 3 × 300 s, 4 × 600 s. All reconstructions were performed applying a maximum likelihood estimation method algorithm by four iterations and six subsets resulting in 0.4 × 0.4 × 0.4 mm voxel size corrected for randoms and scatter.

### Static PET image analyses

From the static images, tumor volumes of interests (VOIs) were segmented by applying an automated isocontour tool that included all voxels with > 40%SUV_max_ or by a set threshold of 2.5 SUV carefully excluding the bladder and kidneys (detailed in next paragraph). Within each tumor VOI the following PET parameters were calculated: mean and maximum standardized uptake values (SUV_mean_, and SUV_max_, respectively), metabolic tumor volume (MTV) and total lesion glycolysis (TLG; TLG = SUV_mean_ x MTV). The static analyses were carried out using InterView Fusion software (Mediso, version 3.01).

In oncology in general and for EC patients, a fixed threshold of > 2.5 SUV is typically applied to segment tumors, aiming to omit normal surrounding tissue from the VOIs while including all likely tumor voxels. By applying this threshold to our cohort we were able to segment tumor in > 95% of the scans; however, the derived VOIs did not include all apparent tumor tissue (See Additional file 1). We measured the mean liver uptake in our PET mice cohort to 0.53 ± 0.06 SUV on average (See Additional file 2), which is substantially lower than the 2.0–3.0 SUV_mean_ reported for human livers [23]. Consequently, we decided to threshold at values 40% of SUV_max_. This led to an average threshold of 1.6 SUV (Additional file 2), which was also more in line with the visual impression of tumor boundaries based on PET and MRI and yielded more similar ratios of vMRI to MTV to that observed in human EC cohorts [24] (See Additional file 3).

### Dynamic PET image analyses

The individual tumor VOIs from the static images were further used as input regions for the dynamic analyses generating tumor time-activity curves in PMOD software (Version 3.8).

To generate the arterial input function (AIF) needed for absolute quantitative modeling of dynamic imaging, we placed a cube-shaped VOI covering the vena cava and selected the seven hottest voxels therein to generate the AIF for each mouse [25, 26]. The shape of each AIF was visually inspected prior to further analyses. The images were analyzed using the kinetic modeling tool (PKIN)-package of PMOD (Version 3.8), extracting the tumor net influx constant (K_i_) by applying the Patlak linear model [27]. We used 0.6 as lumped constant [28] and assumed equal blood glucose level for all mice (6.0 mmol/l) based on previous low intra-animal variation of blood glucose measurements in fasted EC PDX implanted in NSG mice. All fits resulted in < 10% standard error. Tumor metabolic rate of glucose (MR_FDG_) was calculated by the equation MR_FDG_ = K_i_ (blood glucose/lumped constant) [27].

### Histological analyses

To ensure best possible matching of excised tumor tissue to MRI, animals were euthanized immediately after the last imaging. Hematoxylin and eosin (HE) slides (4 μm) of formalin fixed paraffin-embedded tumor tissue were scanned at 20X using a slide scanner (VS120, Olympus). Automatic counting of nuclei was done using the free QuPath (V0.2.0) software [29] in 3–4 rectangular regions of interest (number depending on tumor size) covering the tumor area.

### Statistical analyses

Linear regression analyses for the image-derived tumor markers were performed to examine a possible relationship between the anatomic (MRI) and metabolic (PET) imaging features, between the dynamic- and static PET parameters, and between the anatomic tumor volume (vMRI) and diffusion restriction (ADC_mean_). Differences in tumor markers between the treatment- and control groups were assessed using a Mann–Whitney test. Normality was tested for all variables using Shapiro–Wilk test. P-values were considered to indicate statistical significance when < 0.05. Analyses were done using GraphPad Prism version 9.0.

## Results

### Imaging characteristics of the tumor in the mouse model versus the donor patient

Preoperative pelvic MRI (Fig. 1A–C) and ^18^F-FDG-PET-CT (Fig. 1D and E) in the donor woman with grade 3, endometrioid EC, FIGO stage IIIC1, exhibit tumor characteristics that are shared by the uterine tumor of the derived animal model (Fig. 1F–J). The T2-weighted images (Fig. 1A and F) depict a slightly hyperintense uterine tumor in the patient (Fig. 1A) and an even more hyperintense uterine tumor in the mouse (Fig. 1F); and both tumors have heterogenous signal intensities. Furthermore, both tumors exhibit restricted diffusion with hyperintensity on the DWI b1000 images (Fig. 1B and G) and corresponding hypointensity on the ADC-maps (Fig. 1C and H). Similarly, the uterine tumors are highly ^18^F-FDG-avid on ^18^F-FDG-PET-CT both in the patient (Fig. 1D and E) and the mouse (Fig. 1I and J).

### MRI for monitoring of tumor growth and restricted diffusion

**Fig. 2:**
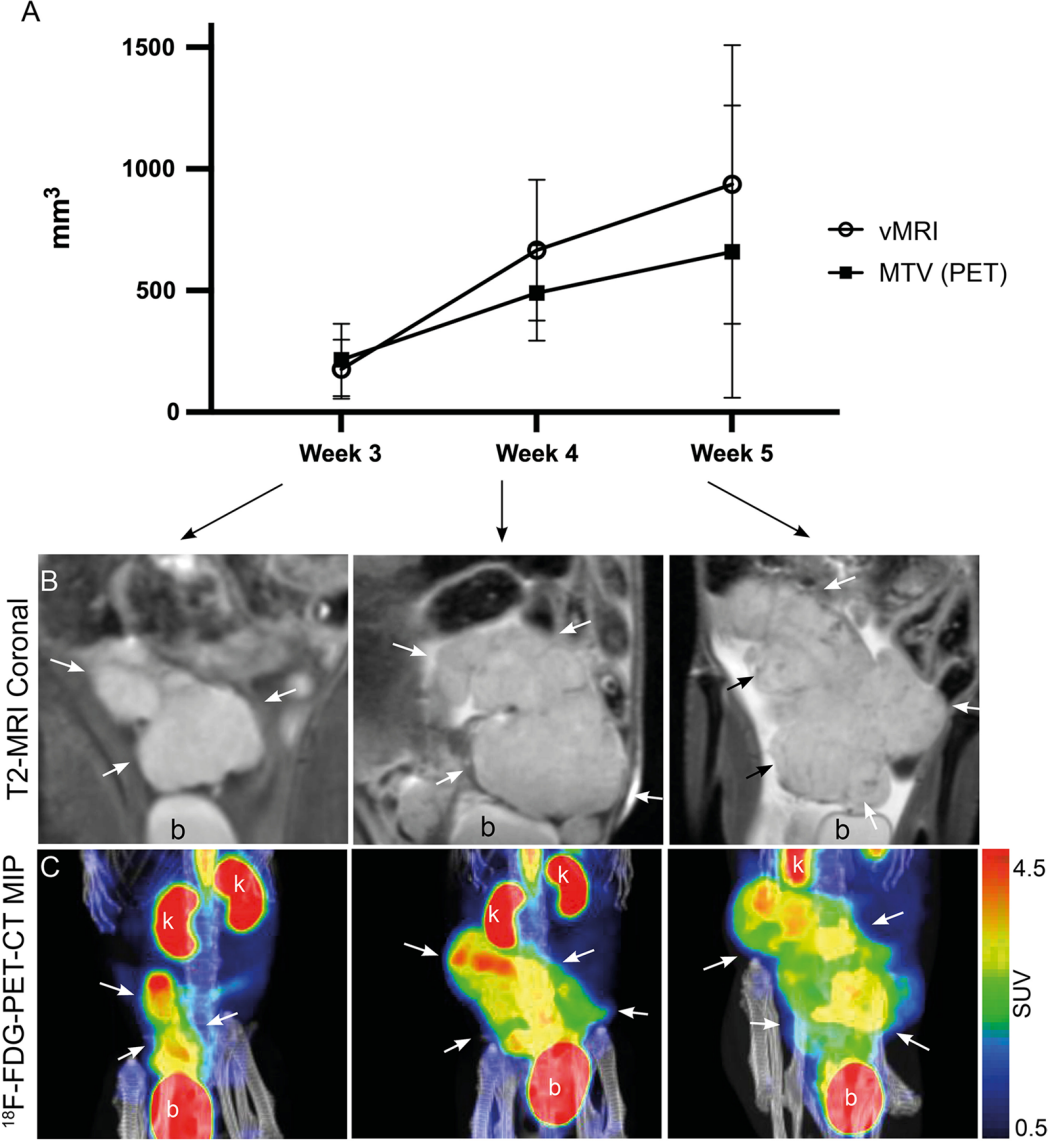
Longitudinal monitoring of tumor growth by MRI and ^18^F-FDG-PET. Upper panel **A** Graph displaying weekly tumor volumes (mean, standard deviation) from T2-weighted MRI (vMRI) and metabolic tumor volume (MTV) from ^18^F-FDG-PET imaging in non-treated mice. The graph is based on imaging of 12 mice in Week 3 (14 mice for MRI), 10 mice in Week 4 and four mice (eight mice for MRI) in Week 5. **B** (T2-MRI) and **C** (^18^F-FDG-PET-CT maximum intensity images, MIP) display the growth of a tumor (arrows) imaged weekly by MRI and PET in a single, representative mouse. *b* bladder, *k* kidney, ^*18*^*F-FDG* fluorodeoxyglucose, *MTV* metabolic tumor volume, *SUV* standardized uptake value, *vMRI* tumor volume from MRI

Tumors were detected by T2-weighted MRI in all mice 3 weeks post-implantation. The estimated tumor volumes (vMRI) increased during Week 3–5 post-implantation with mean (range) vMRI = 177 mm^3^ (2–403) in Week 3 (n = 14), vMRI = 666 mm^3^ (158–1075) in Week 4 (n = 10) and vMRI = 936 mm^3^ (192–1707) in Week 5 (n = 8) (Fig. 2, B). Mean (range) vMRI estimated on all scans acquired Weeks 3–5 (32 scans) was 519 (2–1707) mm^3^ (Table 2). Individual vMRI-values for each mouse are shown in Additional file 4. At DWI (Fig. 1, G and H) the tumors uniformly exhibited restricted diffusion with a mean (range) tumor ADC_mean_-value of 1.07 (0.86–1.48) × 10^–3^ mm^2^/s (Table 2), extracted from whole-volume tumor segmentations on the ADC maps (n = 9) 3–8 weeks post-implantation. The 95% confidence intervals of the means are listed in Table 2.

**Table 2:** Mean values, range and 95% confidence intervals (CI) for ^18^F-FDG-PET and MRI tumor parameters during tumor progression

	SUV_max_	SUV_mean_	MTV (mm^3^)	TLG (SUV_mean_ x MTV)	MR_FDG_ (μmol/min)	ADC_mean_ (10^3^ mm^2^/s)	vMRI (mm^3^)
Scans	26	26	26	26	24	9^a^	32
Mean	3.9	2.2	389	847	0.39	1.07	519
Range [min, max]	[2.1–5.2]	[1.5–2.8]	[49–1271]	[73–2389]	[0.12–0.61]	[0.86–1.48]	[2–1707]
95% CI [lower, upper]	[3.7–4.2]	[2.1–2.3]	[263–515]	[585–1108]	[0.34–0.45]	[0.94 -1.20]	[355–684]

*CI* confidence interval, *SUV* standardized uptake value, *MTV* metabolic tumor volume, *TLG* total lesion glycolysis, *MR*_*FDG*_ metabolic rate of fluorodeoxyglucose (obtained from dynamic imaging), *vMRI* anatomic tumor volume from MRI, *ADC* apparent diffusion coefficient

^a^Includes the four scans from the control mice from the treatment study

### ^18^F-FDG-PET for monitoring tumor metabolism and quantification of tumor metabolic features

**Fig. 3:**
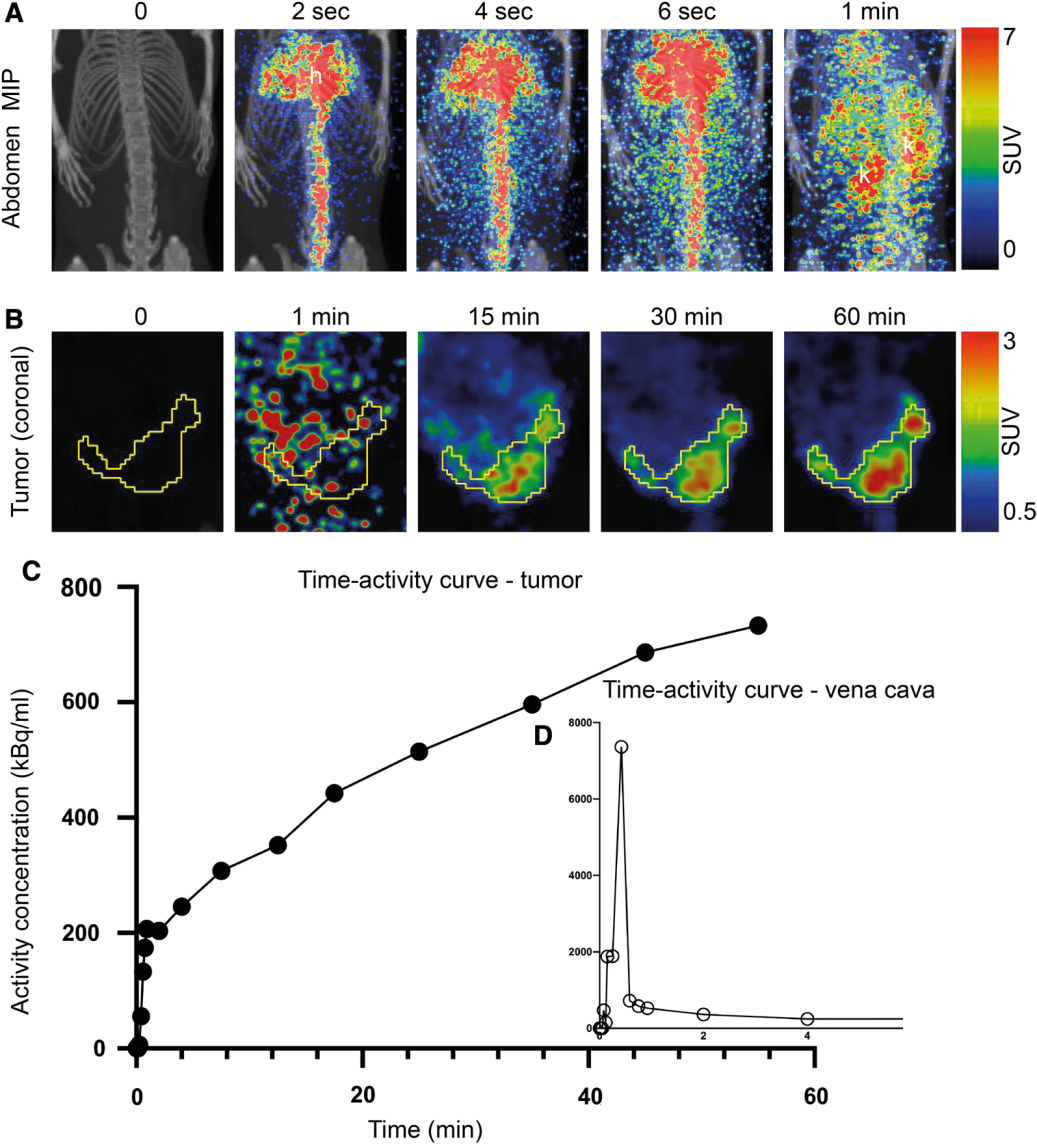
Dynamic ^18^F-FDG-PET imaging. Upper panel **A** Abdominal maximum intensity projection (MIP) images showing the rapid inflow of ^18^F-FDG through the vena cava to the heart (h) and kidneys (k) after an ^18^F-FDG bolus intravenous injection. Middle panel **B** Coronal view of the lower abdomen displaying accumulation of tracer in the tumor (encircled with a yellow ROI) throughout the 1-h dynamic scan, quantified as the time-activity curve of the tumor (**C**) and the image-derived input function quantified from vena cava (insert) (**D**). ^*18*^*F-FDG* fluorodeoxyglucose, *ROI* region of interest, *SUV* standardized uptake value

All ^18^F-FDG-PET scans (26 total) depicted FDG-avid primary uterine tumors (Fig. 1I and J and Fig. 2C). The estimated mean (range) MTV increased during Week 3–5 post-implantation with MTV = 215 mm^3^ (49–366) in Week 3 (n = 12), to MTV = 490 mm^3^ (65–767) in Week 4 (n = 10) and MTV = 660 mm^3^ (117–1271) in Week 5 (n = 4) (Fig. 2C). Altogether, the lesions (scans = 26) had the following mean (range) tumor values for the derived metabolic markers: SUV_mean_ = 2.2 (1.5–2.8), SUV_max_ = 3.9 (2.1–5.2), MTV = 389 (49–1271) mm^3^ and TLG = 847 (73–2389) (Table 2). Individual MTV values for each mouse are shown in Additional file 5. The dynamic series (24 scans) displayed rapid influx of tracer in the vena cava following the ^18^F-FDG bolus injection (Fig. 3A and D) and the tumors characteristically had a rapid accumulation of ^18^F-FDG during the first 20 min, followed by a slow increase in ^18^F-FDG activity during the consecutive 40 min (Fig. 3B and C). The tumor metabolic rate MR_FDG_, non-invasively calculated from the dynamic scans using vena cava as the image-derived input function, had a mean (range) of 0.39 (0.12–0.61) µmol/min (Table 2). The 95% confidence intervals of the means of all PET image parameters are listed in Table 2.

### Correlation between the image-derived tumor markers

**Fig. 4:**
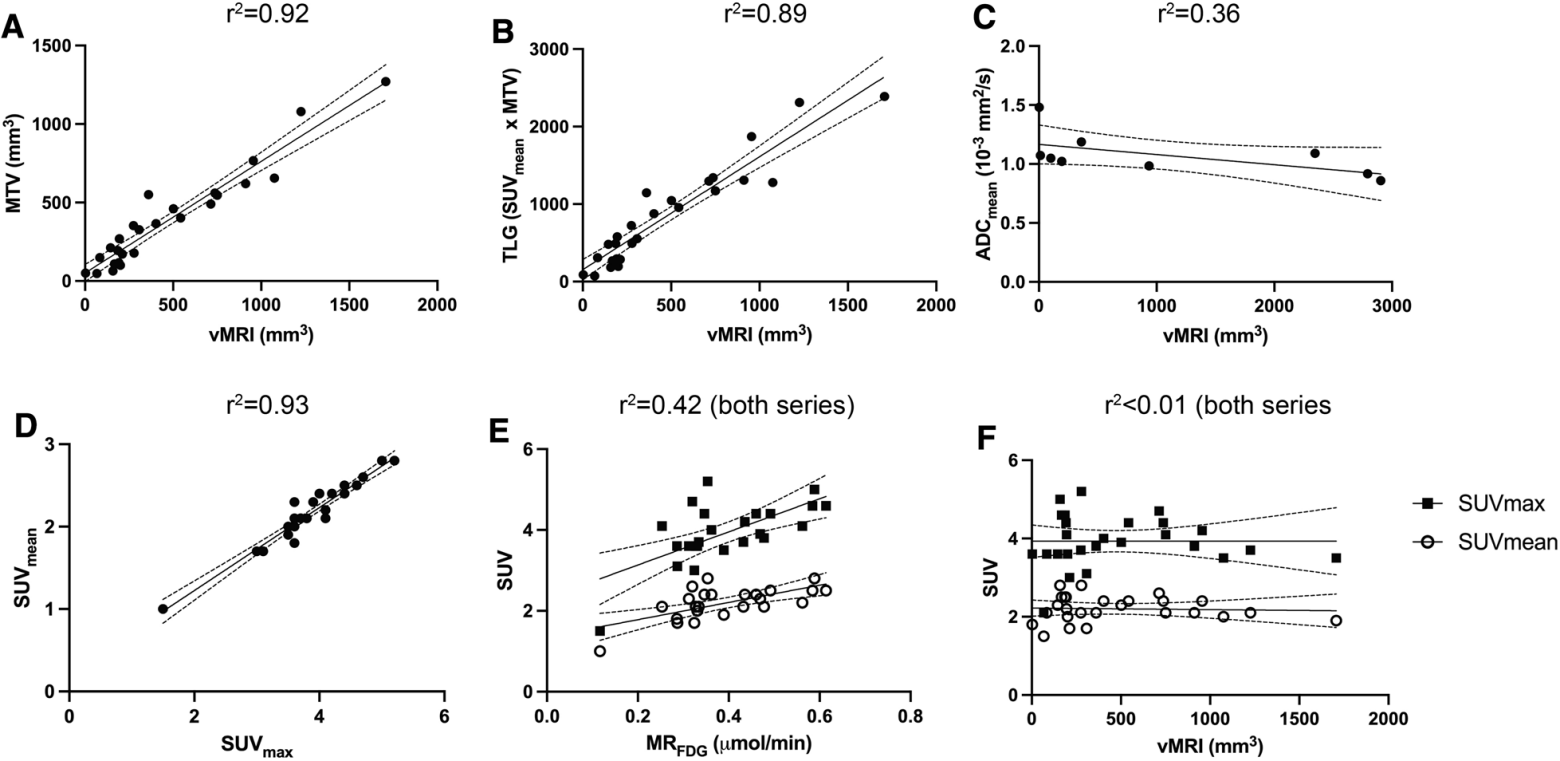
Linear regression analyses of imaging parameters quantified from MRI and ^18^F-FDG-PET. Linear regression analyses of anatomical tumor volume (vMRI) versus metabolic tumor volume (MTV) (**A**), total lesion glycolysis (TLG; SUV_mean_ × MTV) (**B**) and Tumor ADC_mean_ (**C**). Linear regression analyses of SUV_mean_ versus SUV_max_ (**D**), metabolic rate of ^18^F-FDG (MR_FDG_) versus SUV_max_/SUV_mean_ (**E**) and vMRI versus SUV_mean_/_max_ (**F**). Graphs are based on data presented in Table 2. Each dot represents a scan, and the line represents linear regression, with dotted bands representing the 95% confidence bands. r^2^ = goodness of fit (linear regression). *ADC* apparent diffusion coefficient, ^*18*^*F-FDG* fluorodeoxyglucose, MR_FDG_ metabolic rate of ^18^F-FDG, *MTV* metabolic tumor volume, *SUV* standardized uptake value, *TLG* total lesion glycolysis, *vMRI* tumor volume from MRI

For imaging markers acquired during Week 3–5 there was a strong positive linear association between vMRI and both MTV and TLG (TLG = SUV_mean_ × MTV) (r^2^ = 0.92 and 0.89, respectively) (Fig. 4A and B). The standard PET parameters SUV_max_ and SUV_mean_ were strongly positively associated (r^2^ = 0.93) (Fig. 4D) whereas MR_FDG_ was moderately positively associated with SUV_max_ and SUV_mean_ (r^2^ = 0.42 for both) (Fig. 4, E). Tumor ADC_mean_ had a moderate negative association with vMRI (r^2^ = 0.35) (Fig. 4C), whereas no linear association was observed between SUV_mean_/_max_ and vMRI (Fig. 4F). Similar statistical correlations between imaging markers were observed in weekly comparison of the same imaging markers using Spearman correlation-test (Additional file 6).

### Tumor ADC, vMRI and cellular density after chemotherapy

**Fig. 5:**
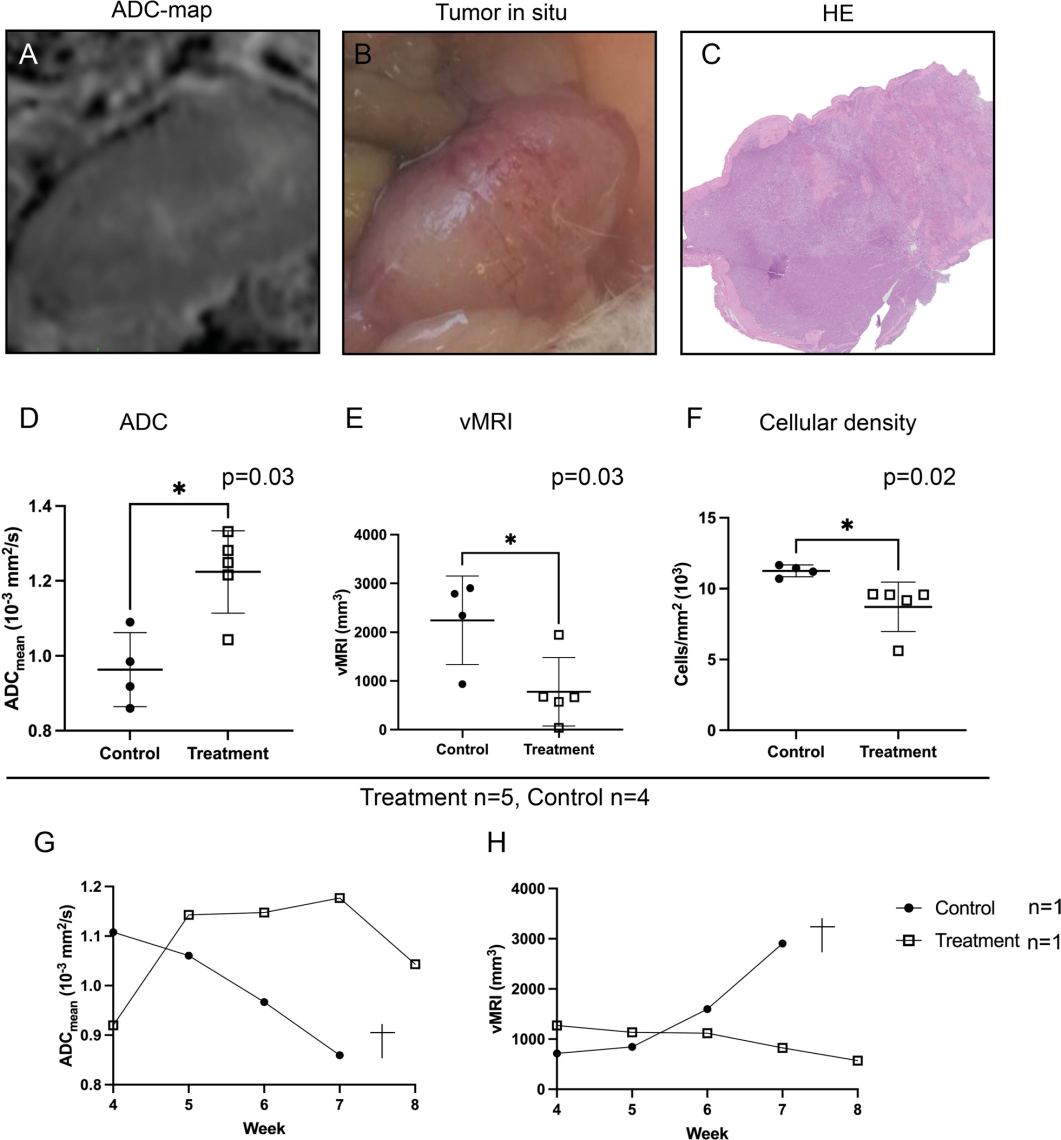
Tumor ADC_mean_, vMRI and cellular density after chemotherapy. ADC-map depicting a large tumor of the left uterine horn (**A**), photograph of tumor in situ immediately after MRI scanning, prior to excision (**B**) and histology section through a representative central part of tumor stained with HE (**C**). A-C are from the same mouse. Tumor ADC_mean_ was significantly higher in treated animals compared to controls (**D**), the tumor volume (vMRI) (**E**) and the tumor cellular density (**F**) was significantly lower in the treated animals compared to the controls. Lines represent mean ± SD, and each dot represents a single sample. Significance was tested using a Mann–Whitney test. Longitudinal data for tumor ADC_mean_ (**G**) and vMRI (**H**) is plotted for one mouse from each group. *ADC* apparent diffusion coefficient, *vMRI* tumor volume from MRI

Mice in the treatment group (n = 5) with DWI prior to sacrifice, had mean (range) tumor ADC_mean_ of 1.2 (1.0–1.3) × 10^–3^ mm^2^/s, which were significantly higher than the mice in the control group (n = 4) having mean (range) tumor ADC_mean_ of 1.0 (0.9–1.1) × 10^–3^ mm^2^/s (p = 0.03) (Fig. 5A, D). Mean vMRI was significantly lower in the treated mice [vMRI = 779 (range, 38–1947) mm^3^] compared to the controls [vMRI = 2245 (935–2905) mm^3^; p = 0.03] (Fig. 5E). Furthermore, the tumor HE-sections at sacrifice displayed significantly lower cellular densities for treated tumors [mean(range) = 8.7(5.6–9.6) × 10^3^ cells/mm^2^] than for untreated tumors [mean = 11.3 (10.7–11.7) × 10^3^ cells/mm^2^; p = 0.02] (Fig. 5F).

One mouse from each group were longitudinally monitored by weekly T2-weighted imaging and DWI, starting at week 4 (one-week after start of treatment) (Fig. 5G and H). At Week 4, the tumor ADC_mean_-value was lower for the treated mouse (0.92 × 10^–3^ mm^2^/s) compared to the control mouse (1.11 × 10^–3^ mm^2^/s), whereas the vMRI was slightly larger in the treated mouse (1272 mm^3^) compared to the control mouse (714 mm^3^). The tumor ADC_mean_-values in the treated mouse increased during treatment (from 0.92 to 1.04 × 10^–3^ mm^2/^s) indicating normalization of the restricted diffusion observed prior to treatment, whereas the control mouse had a gradual decrease (from 1.11 to 0.86 × 10^–3^ mm^2^/s) in tumor ADC_mean_ indicating increased diffusion restriction. Similarly, the vMRI for the treatment mouse was gradually decreasing (from 1272 to 571 mm^3^ at endpoint) indicating treatment response, whereas the mouse in the control group had rapidly increasing tumor vMRI (from 714 to 2905 mm^3^ at endpoint) and had to be sacrificed prior to end of study due to high disease burden.

## Discussion

In this study we demonstrate that advanced MRI and PET imaging methods in preclinical EC allow non-invasive and quantitative monitoring of tumor progression and treatment response. To our knowledge, this is the first study demonstrating the feasibility of a preclinical imaging platform, mirroring imaging methods widely employed in the clinic, for characterization of clinically relevant orthotopic EC mouse models.

We found a strong positive linear relationship between vMRI and MTV and a slightly negative linear relationship between vMRI and tumor ADC, indicating increased glycolysis and cellularity in tumors with large volume. Similarly, preoperative imaging studies in high-grade EC patients also report that vMRI and MTV are positively correlated [24], and that vMRI and ADC are negatively correlated [17, 24, 30], supporting the high clinical relevance and potential translatability of our preclinical model. In our study, no evident relationship between vMRI and SUV_mean_/SUV_max_ was observed. This finding is different from that reported in a recent large clinical EC study (n = 215) finding strong positive correlations between vMRI and SUV_mean_/_max_ (Spearman r = 0.61/0.56) though even stronger positive correlation was reported between vMRI and MTV [24]. Importantly, the findings in this preclinical EC study suggests that the commonly reported PET parameters SUV_mean_/_max_ cannot substitute vMRI or MTV for assessing tumor burden in preclinical models. However, more preclinical studies are needed to explore whether the observed lack of correlation between SUV _mean_/_max_ and tumor volume is the same for all EC models. Furthermore, segmentation methods and threshold values should be systematically evaluated as the current use of these are highly variable in the literature.

This study is the first to present data from dynamic ^18^F-FDG-PET imaging (MR_FDG_) in an orthotopic EC PDX model. The MR_FDG_ values were similar to that reported for subcutaneous breast- and brain cell line tumors in preclinical studies [31]. Moreover, tumor MR_FDG_ values from a large cohort including 11 different cell line tumors were in the same range as ours [32]. Interestingly, a recent dynamic ^18^F-FDG-PET study including 101 patients diagnosed with a range of cancers, demonstrated clinical feasibility and superior quantification using MR_FDG_ with higher tumor-to-background- and contrast-to-noise ratios compared to conventional tumor SUV values [20]. Interestingly, they reported a significant positive correlation between K_i_ (which is directly derived from MR_FDG_) and SUV_mean_, which is in line with our finding.

We demonstrate the usefulness of T2-weighted MRI for non-invasive monitoring of tumor volume in mice treated with paclitaxel and carboplatin, which is the standard adjuvant chemotherapy for EC [33]. In line with our study, T2-weighted MRI has been used to monitor tumor volume reduction after monotherapy with rapamycin in a genetically engineered mouse model of EC (Lkb1-deficient) [34] and after combined treatment with Olaparib and a PI3K-inhibitor (BKM120) in PTEN-deficient endometrioid EC model [35]. We additionally included DWI at the end of the experiment to explore tumor cellularity in the treatment-versus the control group. Treatment-induced cell death is known to normalize tumor cellularity and tumor microstructure, making it more similar to that of nonmalignant tissues; this effect can be detected by increased tumor ADC values [36]. Our longitudinal imaging data showcased restricted diffusion prior to treatment, followed by increased tumor ADC_mean_ during treatment. The increase in ADC_mean_ was evident prior to tumor volume reduction on the T2-weighted images. This may suggest that tumor ADC_mean_ is a powerful imaging parameter for early detection of treatment response preceding the decrease in tumor volumes depicted by T2-weighted anatomical series.

To our knowledge, no previous preclinical studies have utilized DWI and ADC to assess treatment response in EC. However, early increase in tumor ADC values has been reported in subcutaneous ovarian cancer xenografts 3 days post-treatment with a PI3K/mTOR-inhibitor [37], a pathway of therapeutic interest also in EC [38]. Similarly, increased tumor ADC values 24 h after radiotherapy (20 Gy) has been shown in subcutaneous U14 cervical allografts, also prior to changes in tumor volume [39]. Unfortunately, in the present study the COVID-19 lockdown precluded PET-imaging in treated mice; thus, we were not able to compare potential response markers, static or dynamic, from ^18^F-FDG-PET with that from MRI. In a previous preclinical EC treatment study (with treatment groups: paclitaxel, trastuzumab or controls) imaged by ^18^F-FDG-PET at study endpoint, similar tumor SUV_mean_ was found for treated mice and controls and also similar tumor weights at the end of the experiment [21]. Wang et al. recently reported significant decrease in SUV_max_ values in lung metastases from a cell line-based EC model following treatment with an inhibitory PI3K-pathway agent [40]. Thus, future studies are needed to establish the optimal role of imaging markers from ^18^F-FDG-PET and MRI for monitoring treatment response in preclinical EC models.

Although increased tumor metabolism (relative to normal surrounding tissue) is clearly evident by ^18^F-FDG-PET imaging in our preclinical orthotopic EC cohort, the mean SUV_max_/SUV_mean_ of 3.9/2.2 is lower than that reported for human primary EC (SUV_max_/SUV_mean_ median of 14.1/5.4 [24]). This is not surprising given the differences in ^18^F-FDG metabolism, employed segmentation threshold and fasting period prior to imaging in our preclinical setting compared to the clinical setting. Interestingly, the tumor ADC_mean_ values in our mouse cohort (mean 1.07 × 10^–3^ mm^2^/s) were more similar to that typically reported for human tumors (median 0.78 × 10^–3^ mm^2^/s in a recent EC study [24]). Thus, the combination of utilizing our relevant organoid model and preclinical DWI seems to very well reproduce the microstructural tumor features (reflected in ADC values) observed in human EC.

Several tumor segmentation methods have been developed in PET, including both manual-, boundary- and region-based techniques and the chosen approach will inherently impact the calculated parameter outputs [41]. Deploying two commonly used clinical segmentation methods yielded largely different results for MTV in our study. Future preclinical PET studies should ideally assess multiple segmentation methods in order to determine the optimal approach for valid tumor segmentations in that particular study. Which segmentation algorithm that is preferable will depend on various factors including type of mouse model, fasting protocol, PET tracer, disease type as well as other physiological factors.

Our study has some limitations. A combined MRI and PET scanner would have been beneficial in this study since it would allow more accurate co-registration of anatomic tumor volumes and a detailed comparison of morphologic- and functional tumor features in the same voxels. However, as small-animal hybrid PET-MRI scanners are becoming more common, this opens the avenue for utilizing this novel imaging platform in the future. Dynamic quantitative PET imaging requires an arterial input function (AIF) which in preclinical studies can be challenging to obtain with the gold standard of blood sampling, since mice have small blood volumes [6]. Hence, we used an image-derived input function from vena cava that allowed noninvasive and longitudinal analyses. Studies have shown that an image-derived input function using the vena cava in small animals is an accurate method for obtaining the AIF [25, 26]. However, our input function has not been corrected for partial volume effect nor experimentally validated. Additionally, former studies show that MR_FDG_ is highly dependent on blood glucose levels [31, 32]. In our study, the blood glucose was set to 6.0 mmol/ for all mice based on low intra-animal variation in previously measured values in the same mouse strain, tumor type and fasting protocol. Thus, our choice of using a fixed blood glucose level for modelling, may potentially have led to both over- and underestimation of the calculated MR_FDG_ in the present study.

Finally, the whole tumor ADC_mean_-values in this study were compared to tumor cellular density quantified from a single histology section. Using whole tumor segmentation rather than single or multiple regions of interest to calculate ADC_mean_-values removes the potential selection bias regarding placement of the ROIs. Nevertheless, we are comparing data extracted from 3D (ADC) with data from 2D (cellular density), the latter being potentially less representative of the entire tumor volume. This limitation is, however, typically shared in a clinical patient setting, and where exact co-registration of preoperative MRI images with tissue slices from hysterectomy specimen is very difficult to achieve.

## Conclusions

We have demonstrated the feasibility of advanced MRI- and PET imaging methods in a preclinical organoid EC model for monitoring tumor size, microstructural- and metabolic features during tumor progression. Following treatment with standard chemotherapy, ADC-values from DWI-MRI may represent a powerful imaging marker for detecting early treatment response. Relevant imaging platforms, mirroring imaging methods widely employed in the clinical diagnostic work-up, should be utilized in future preclinical studies in order to enhance the potential for clinical translatability and add momentum to the development of new imaging-guided therapeutic strategies in EC.

## Supplementary Information

**Additional file 1. ** PET tumor segmentation. Two different PET tumor segmentation algorithms illustrated on a coronal PET-CT slice (A) displaying the lower abdomen and the bladder (b). The tumor was either outlined using a fixed threshold of 2.5 to include putative tumor voxels in the red VOI (MTV = 93 mm3) or by using a 40% of the tumor SUVmax (40% SUVmax) shown by the yellow VOI (MTV = 561 mm3). This specific tumor had SUVmax of 3.8 thus the segmentation threshold was 1.5 for this example. The matched coronal T2-weighted MRI (B) displays the tumor (white arrows) and bladder (b). The vMRI for this tumor was 403 mm3. We chose the 40% SUVmax -segmentation method for the present study. Abbreviations; MTV = metabolic tumor volume, SUV = standardized uptake value, vMRI = tumor volume from MRI, VOI = volume of interest.

**Additional file 2. ** Overview of measured SUV_mean_ values in liver and threshold-values used for tumor segmentation.

**Additional file 3. ** Calculated mean metabolic tumor volumes (MTV) using the two alternative segmentation algorithms and corresponding mean anatomic tumor volumes from MRI (vMRI).

**Additional file 4. ** Individual and longitudinal vMRI values for all mice.

**Additional file 5. ** Individual and longitudinal MTV values for all mice.

**Additional file 6. ** Weekly correlation of imaging parameters quantified from MRI and 18F-FDG-PET. Correlation of imaging parameters in weeks 3, 4 and 5 after tumor implantation. Spearman correlation (r) and p-values are indicated for each plot, in addition to the r2-value to display goodness-of-fit for linear regression. 18F-FDG = fluorodeoxyglucose, MRFDG = metabolic rate of 18F-FDG, MTV = metabolic tumor volume, r = Spearman correlation, r2 = goodness-of-fit, linear regression, SUV = standardized uptake value, TLG = total lesion glycolysis, vMRI = tumor volume from MRI

## Data Availability

The datasets used and/or analysed during the current study are available from the corresponding author on reasonable request.

## Abbreviations

ADC: Apparent diffusion coefficient
AIF: Arterial input function
DWI: Diffusion-weighted MRI
EC: Endometrial cancer
^18^F-FDG: Fluorodeoxyglucose
MR_FDG_: Metabolic rate of ^18^F-FDG
MRI: Magnetic resonance imaging
MTV: Metabolic tumor volume
O-PDX: Organoid-based patient-derived xenograft
PET: Positron emission tomography
ROI: Region of interest
SUV: Standardized uptake value
TLG: Total lesion glycolysis
vMRI: Tumor volume from MRI
VOI: Volume of interest

## References

[1] Arrowsmith J, Miller P (2013). Phase II and phase III attrition rates 2011–2012. Nat Rev Drug Discov.

[2] de Jong M, Essers J, van Weerden WM (2014). Imaging preclinical tumour models: improving translational power. Nat Rev Cancer.

[3] Ben-David U, Siranosian B, Ha G, Tang H, Oren Y, Hinohara K, Strathdee CA, Dempster J, Lyons NJ, Burns R, Nag A, Kugener G, Cimini B, Tsvetkov P, Maruvka YE, O’Rourke R, Garrity A, Tubelli AA, Bandopadhayay P, Tsherniak A, Vazquez F, Wong B, Birger C, Ghandi M, Thorner AR, Bittker JA, Meyerson M, Getz G, Beroukhim R, Golub TR (2018). Genetic and transcriptional evolution alters cancer cell line drug response. Nature.

[4] Moiola CP, Lopez-Gil C, Cabrera S, Garcia A, Van Nyen T, Annibali D, Fonnes T, Vidal A, Villanueva A, Matias-Guiu X, Krakstad C, Amant F, Gil-Moreno A, Colas E (2018). Patient-derived xenograft models for endometrial cancer research. Int J Mol Sci.

[5] Van Nyen T, Moiola CP, Colas E, Annibali D, Amant F (2018). Modeling endometrial cancer: past, present, and future. Int J Mol Sci.

[6] Espedal H, Fonnes T, Fasmer KE, Krakstad C, Haldorsen IS (2019). Imaging of preclinical endometrial cancer models for monitoring tumor progression and response to targeted therapy. Cancers.

[7] Berg HF, Hjelmeland ME, Lien H, Espedal H, Fonnes T, Srivastava A, Stokowsky T, Strand E, Bozickovic O, Stefansson IM, Bjørge L, Trovik J, Haldorsen IS, Hoivik EA, Krakstad C (2021). Patient-derived organoids reflect the genetic profile of endometrial tumors and predict patient prognosis. Commun Med.

[8] Haldorsen IS, Popa M, Fonnes T, Brekke N, Kopperud R, Visser NC, Rygh CB, Pavlin T, Salvesen HB, McCormack E, Krakstad C (2015). Multimodal imaging of orthotopic mouse model of endometrial carcinoma. PLoS ONE.

[9] Miller KD, Nogueira L, Mariotto AB, Rowland JH, Yabroff KR, Alfano CM, Jemal A, Kramer JL, Siegel RL (2019). Cancer treatment and survivorship statistics, 2019. CA Cancer J Clin.

[10] Sung H, Ferlay J, Siegel RL, Laversanne M, Soerjomataram I, Jemal A, Bray F (2021). Global cancer statistics 2020: GLOBOCAN estimates of incidence and mortality worldwide for 36 cancers in 185 countries. CA Cancer J Clin.

[11] Amant F, Moerman P, Neven P, Timmerman D, Van Limbergen E, Vergote I (2005). Endometrial cancer. Lancet.

[12] Fung-Kee-Fung M, Dodge J, Elit L, Lukka H, Chambers A, Oliver T (2006). Follow-up after primary therapy for endometrial cancer: a systematic review. Gynecol Oncol.

[13] Lu KH, Broaddus RR (2020). Endometrial cancer. N Engl J Med.

[14] Matei D, Filiaci V, Randall ME, Mutch D, Steinhoff MM, DiSilvestro PA, Moxley KM, Kim YM, Powell MA, O'Malley DM, Spirtos NM, Small W, Tewari KS, Richards WE, Nakayama J, Matulonis UA, Huang HQ, Miller DS (2019). Adjuvant chemotherapy plus radiation for locally advanced endometrial cancer. N Engl J Med.

[15] Galaal K, Al Moundhri M, Bryant A, Lopes AD, Lawrie TA (2014). Adjuvant chemotherapy for advanced endometrial cancer. Cochrane Database Syst Rev.

[16] Haldorsen IS, Salvesen HB (2016). What is the best preoperative imaging for endometrial cancer?. Curr Oncol Rep.

[17] Fasmer KE, Bjørnerud A, Ytre-Hauge S, Grüner R, Tangen IL, Werner HM, Bjørge L, Salvesen ØO, Trovik J, Krakstad C, Haldorsen IS (2018). Preoperative quantitative dynamic contrast-enhanced MRI and diffusion-weighted imaging predict aggressive disease in endometrial cancer. Acta Radiol.

[18] Husby JA, Reitan BC, Biermann M, Trovik J, Bjorge L, Magnussen IJ, Salvesen OO, Salvesen HB, Haldorsen IS (2015). Metabolic tumor volume on 18F-FDG PET/CT improves preoperative identification of high-risk endometrial carcinoma patients. J Nucl Med.

[19] Bollineni VR, Ytre-Hauge S, Bollineni-Balabay O, Salvesen HB, Haldorsen IS (2016). High diagnostic value of 18F-FDG PET/CT in endometrial cancer: systematic review and meta-analysis of the literature. J Nucl Med.

[20] Dias AH, Pedersen MF, Danielsen H, Munk OL, Gormsen LC (2020). Clinical feasibility and impact of fully automated multiparametric PET imaging using direct Patlak reconstruction: evaluation of 103 dynamic whole-body 18F-FDG PET/CT scans. Eur J Nucl Med Mol Imaging.

[21] Fonnes T, Strand E, Fasmer KE, Berg HF, Espedal H, Sortland K, Stefansson I, Bjorge L, Haldorsen IS, Krakstad C, McCormack E (2020). Near-infrared fluorescent imaging for monitoring of treatment response in endometrial carcinoma patient-derived xenograft models. Cancers.

[22] Yushkevich PA, Piven J, Hazlett HC, Smith RG, Ho S, Gee JC, Gerig G (2006). User-guided 3D active contour segmentation of anatomical structures: significantly improved efficiency and reliability. Neuroimage.

[23] Boellaard R, Delgado-Bolton R, Oyen WJ, Giammarile F, Tatsch K, Eschner W, Verzijlbergen FJ, Barrington SF, Pike LC, Weber WA, Stroobants S, Delbeke D, Donohoe KJ, Holbrook S, Graham MM, Testanera G, Hoekstra OS, Zijlstra J, Visser E, Hoekstra CJ, Pruim J, Willemsen A, Arends B, Kotzerke J, Bockisch A, Beyer T, Chiti A, Krause BJ, European Association of Nuclear M (2015). FDG PET/CT: EANM procedure guidelines for tumour imaging: version 2.0. Eur J Nucl Med Mol Imaging.

[24] Fasmer KE, Gulati A, Dybvik JA, Ytre-Hauge S, Salvesen Ø, Trovik J, Krakstad C, Haldorsen IS (2020). Preoperative 18F-FDG PET/CT tumor markers outperform MRI-based markers for the prediction of lymph node metastases in primary endometrial cancer. Eur Radiol.

[25] Lanz B, Poitry-Yamate C, Gruetter R (2014). Image-derived input function from the vena cava for 18F-FDG PET studies in rats and mice. J Nucl Med.

[26] Thackeray JT, Bankstahl JP, Bengel FM (2015). Impact of image-derived input function and fit time intervals on Patlak quantification of myocardial glucose uptake in mice. J Nucl Med.

[27] Patlak CS, Blasberg RG (1985). Graphical evaluation of blood-to-brain transfer constants from multiple-time uptake data. Generalizations. J Cereb Blood Flow Metab.

[28] Lear JL, Ackermann RF (1989). Regional comparison of the lumped constants of deoxyglucose and fluorodeoxyglucose. Metab Brain Dis.

[29] Bankhead P, Loughrey MB, Fernandez JA, Dombrowski Y, McArt DG, Dunne PD, McQuaid S, Gray RT, Murray LJ, Coleman HG, James JA, Salto-Tellez M, Hamilton PW (2017). QuPath: open source software for digital pathology image analysis. Sci Rep.

[30] Husby JA, Salvesen ØO, Magnussen IJ, Trovik J, Bjørge L, Salvesen HB, Haldorsen IS (2015). Tumour apparent diffusion coefficient is associated with depth of myometrial invasion and is negatively correlated to tumour volume in endometrial carcinomas. Clin Radiol.

[31] Sha W, Ye H, Iwamoto KS, Wong KP, Wilks MQ, Stout D, McBride W, Huang SC (2013). Factors affecting tumor (18) F-FDG uptake in longitudinal mouse PET studies. EJNMMI Res.

[32] Williams SP, Flores-Mercado JE, Port RE, Bengtsson T (2012). Quantitation of glucose uptake in tumors by dynamic FDG-PET has less glucose bias and lower variability when adjusted for partial saturation of glucose transport. EJNMMI Res.

[33] Miller D, Filiaci V, Fleming G, Mannel R, Cohn D, Matsumoto T, Tewari K, DiSilvestro P, Pearl M, Zaino R (2012). Randomized phase III noninferiority trial of first line chemotherapy for metastatic or recurrent endometrial carcinoma: a gynecologic oncology group study. Gynecol Oncol.

[34] Contreras CM, Akbay EA, Gallardo TD, Haynie JM, Sharma S, Tagao O, Bardeesy N, Takahashi M, Settleman J, Wong KK, Castrillon DH (2010). Lkb1 inactivation is sufficient to drive endometrial cancers that are aggressive yet highly responsive to mTOR inhibitor monotherapy. Dis Model Mech.

[35] Bian X, Gao J, Luo F, Rui C, Zheng T, Wang D, Wang Y, Roberts TM, Liu P, Zhao JJ, Cheng H (2018). PTEN deficiency sensitizes endometrioid endometrial cancer to compound PARP-PI3K inhibition but not PARP inhibition as monotherapy. Oncogene.

[36] Herneth AM, Guccione S, Bednarski M (2003). Apparent diffusion coefficient: a quantitative parameter for in vivo tumor characterization. Eur J Radiol.

[37] Cebulla J, Huuse EM, Pettersen K, van der Veen A, Kim E, Andersen S, Prestvik WS, Bofin AM, Pathak AP, Bjorkoy G, Bathen TF, Moestue SA (2015). MRI reveals the in vivo cellular and vascular response to BEZ235 in ovarian cancer xenografts with different PI3-kinase pathway activity. Br J Cancer.

[38] Salvesen HB, Haldorsen IS, Trovik J (2012). Markers for individualised therapy in endometrial carcinoma. Lancet Oncol.

[39] Huang Y, Huang J, Feng M, Ren J, Mi K, Cheng J, Song B, Lang J (2017). Early changes in the apparent diffusion coefficient and MMP-9 expression of a cervical carcinoma U14 allograft model following irradiation. Oncol Lett.

[40] Wang Y, Ren F, Li B, Song Z, Chen P, Ouyang L (2019). Ellagic acid exerts antitumor effects via the PI3K signaling pathway in endometrial cancer. J Cancer.

[41] Foster B, Bagci U, Mansoor A, Xu Z, Mollura DJ (2014). A review on segmentation of positron emission tomography images. Comput Biol Med.

