# Supplementary figures and images for: Feasibility and utility of MRI and dynamic 18F-FDG-PET in an orthotopic organoid-based patient-derived mouse model of endometrial cancer
Source: J Transl Med. 2021 Sep 26;19:406. doi: 10.1186/s12967-021-03086-9 (PMC8474962; doi:10.1186/s12967-021-03086-9)

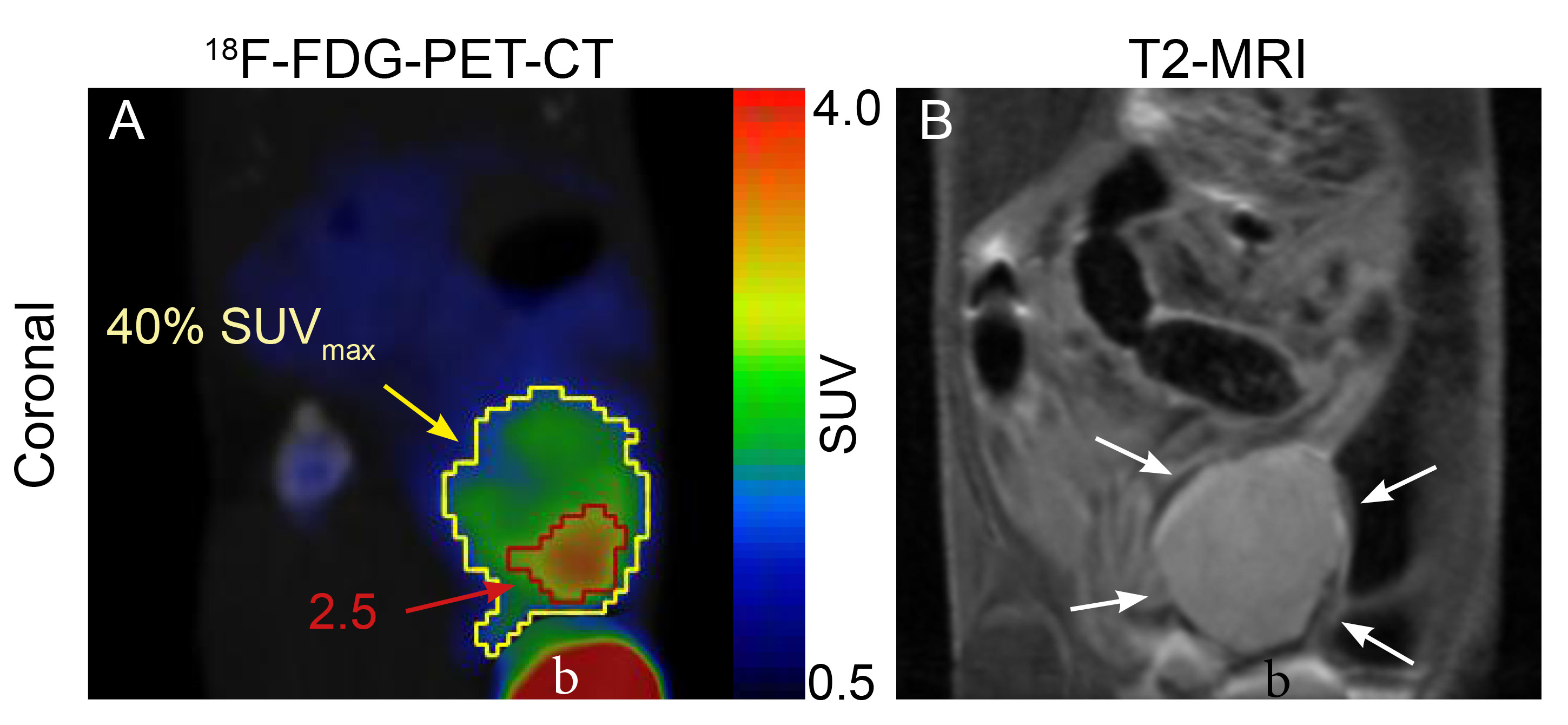

Supplement: Supplementary file 1 — Additional file 1. PET tumor segmentation. Two different PET tumor segmentation algorithms illustrated on a coronal PET-CT slice (A) displaying the lower abdomen and the bladder (b). The tumor was either outlined using a fixed threshold of 2.5 to include putative tumor voxels in the red VOI (MTV = 93 mm3) or by using a 40% of the tumor SUVmax (40% SUVmax) shown by the yellow VOI (MTV = 561 mm3). This specific tumor had SUVmax of 3.8 thus the segmentation threshold was 1.5 for this example. The matched coronal T2-weighted MRI (B) displays the tumor (white arrows) and bladder (b). The vMRI for this tumor was 403 mm3. We chose the 40% SUVmax -segmentation method for the present study. Abbreviations; MTV = metabolic tumor volume, SUV = standardized uptake value, vMRI = tumor volume from MRI, VOI = volume of interest. [file 12967_2021_3086_MOESM1_ESM.jpg]

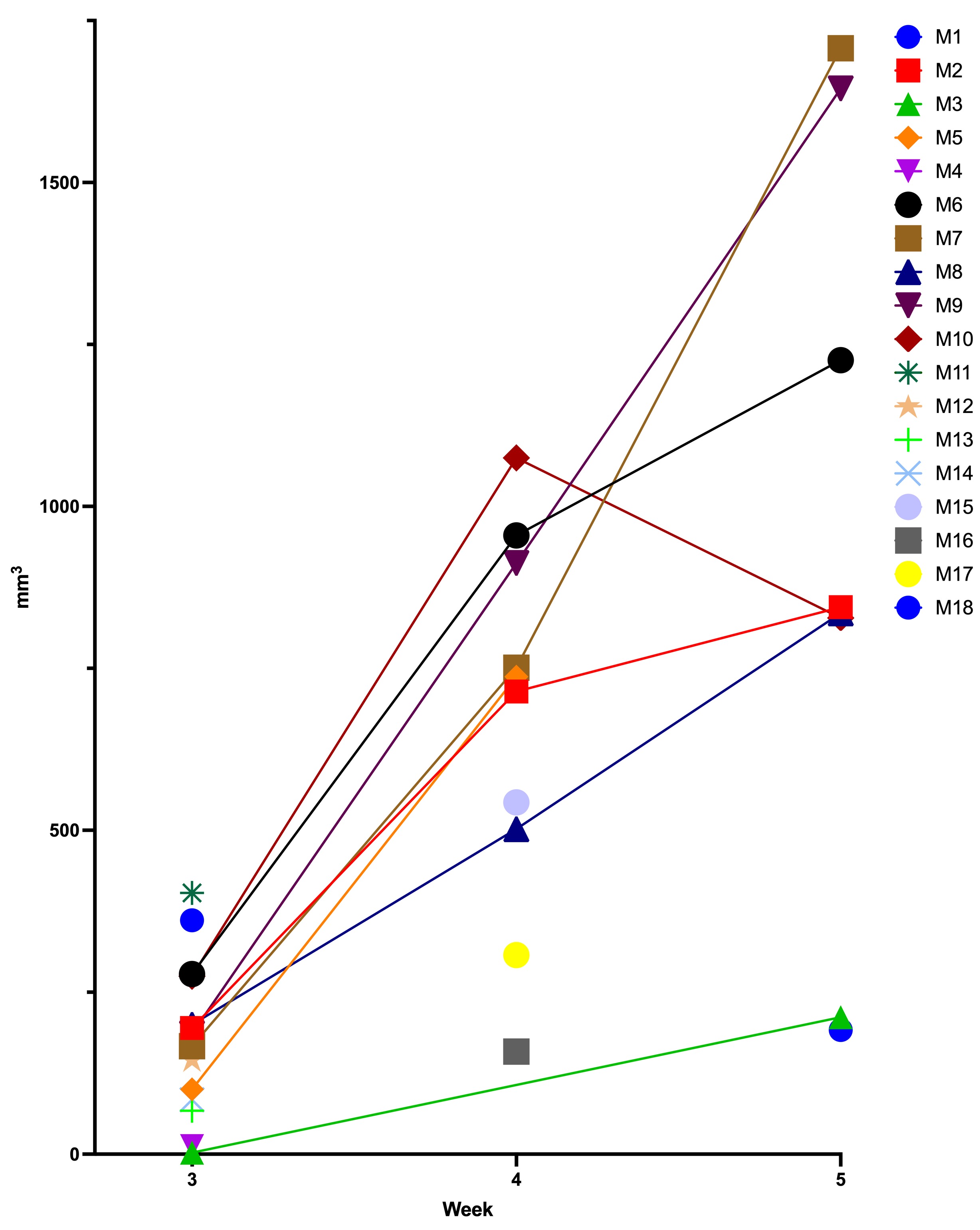

Supplement: Supplementary file 4 — Additional file 4. Individual and longitudinal vMRI values for all mice. [file 12967_2021_3086_MOESM4_ESM.jpg]

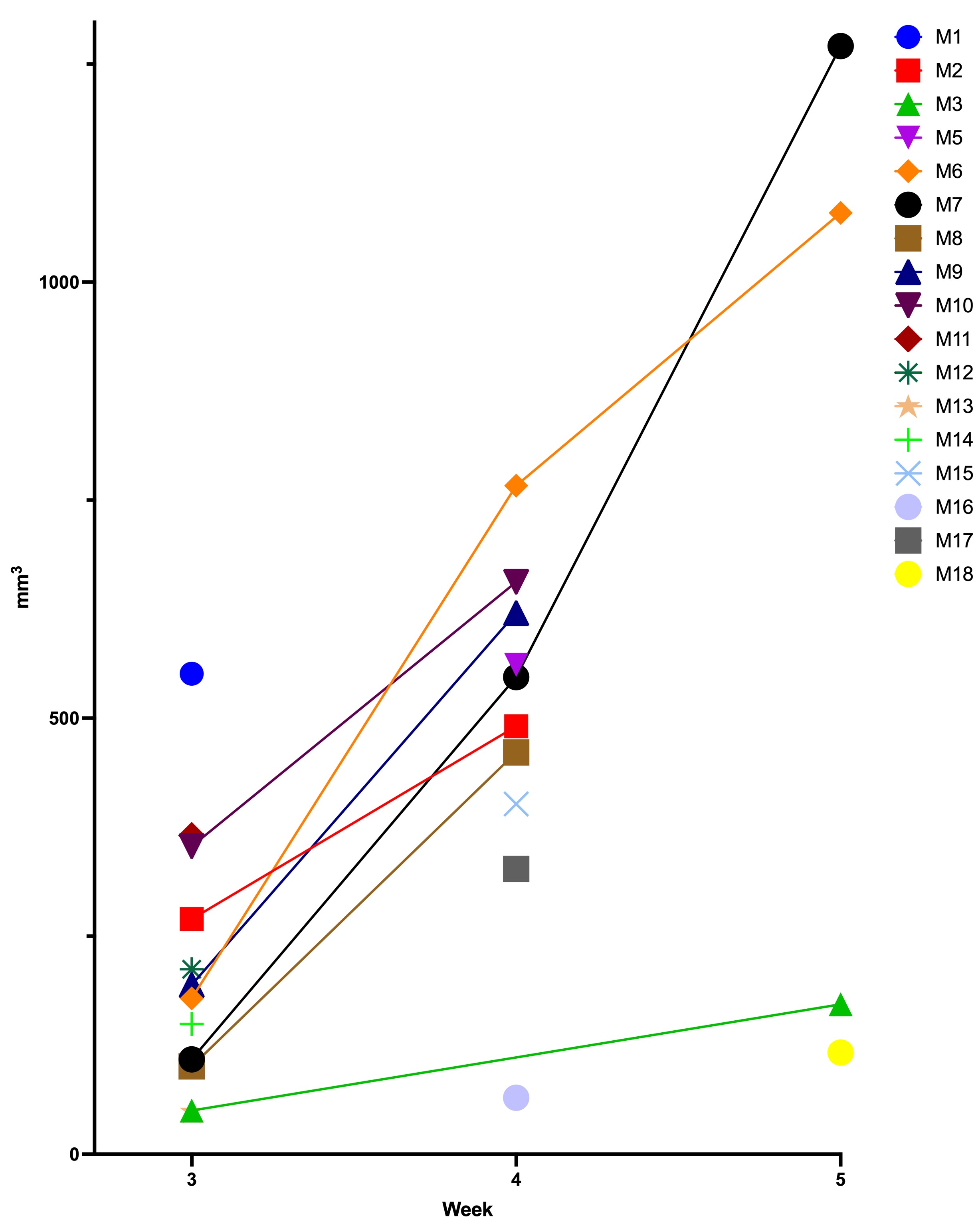

Supplement: Supplementary file 5 — Additional file 5. Individual and longitudinal MTV values for all mice. [file 12967_2021_3086_MOESM5_ESM.jpg]

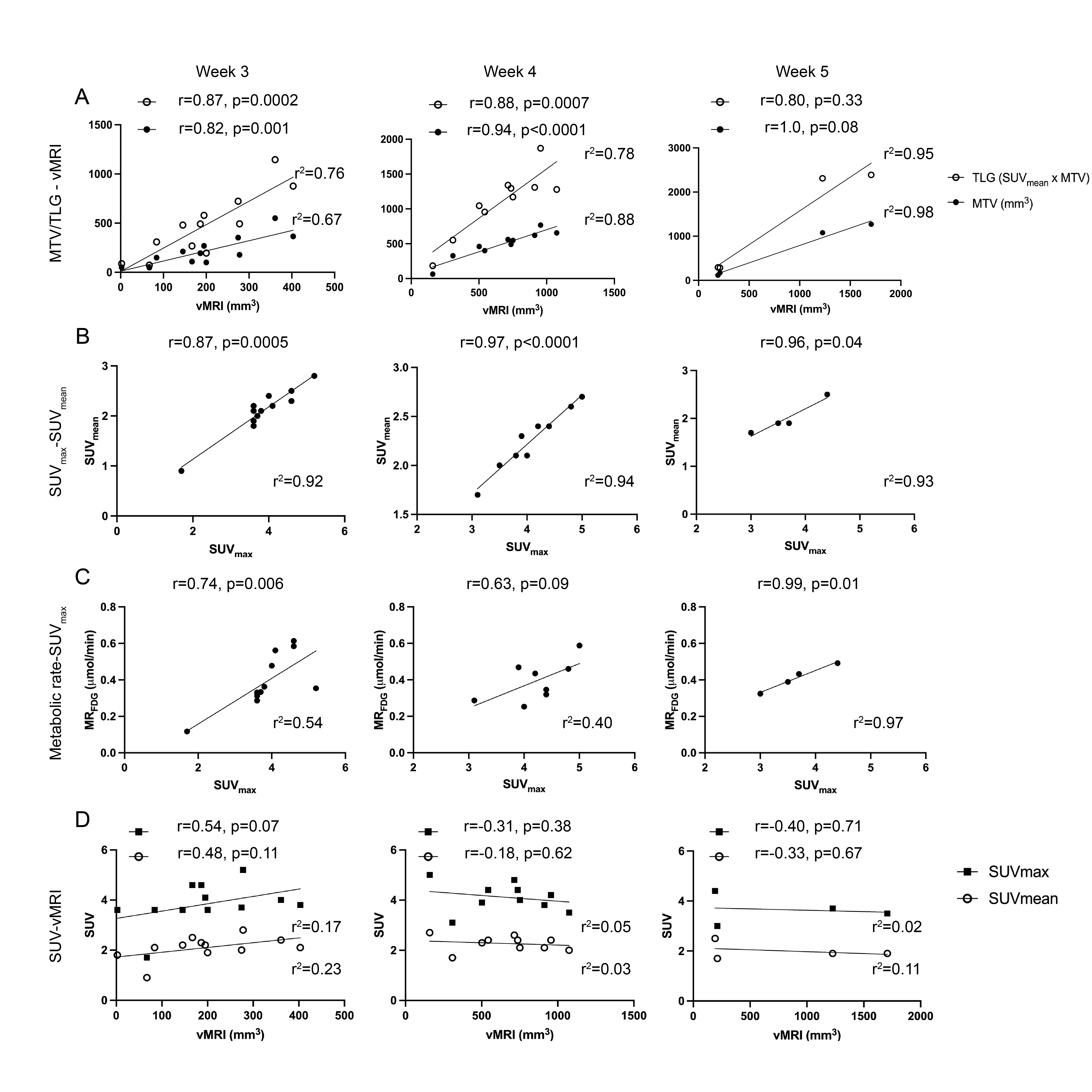

Supplement: Supplementary file 6 — Additional file 6. Weekly correlation of imaging parameters quantified from MRI and 18F-FDG-PET. Correlation of imaging parameters in weeks 3, 4 and 5 after tumor implantation. Spearman correlation (r) and p-values are indicated for each plot, in addition to the r2-value to display goodness-of-fit for linear regression. 18F-FDG = fluorodeoxyglucose, MRFDG = metabolic rate of 18F-FDG, MTV = metabolic tumor volume, r = Spearman correlation, r2 = goodness-of-fit, linear regression, SUV = standardized uptake value, TLG = total lesion glycolysis, vMRI = tumor volume from MRI [file 12967_2021_3086_MOESM6_ESM.jpg]
