# Supplementary material for: Feasibility and utility of MRI and dynamic 18F-FDG-PET in an orthotopic organoid-based patient-derived mouse model of endometrial cancer
Source: J Transl Med. 2021 Sep 26;19:406. doi: 10.1186/s12967-021-03086-9 (PMC8474962; doi:10.1186/s12967-021-03086-9)
Supplement: Supplementary file 2 — Additional file 2. Overview of measured SUVmean values in liver and threshold-values used for tumor segmentation. [file 12967_2021_3086_MOESM2_ESM.docx]

**Additional file 2, title:** Overview of measured SUV_mean_ values in liver and threshold-values used for tumor segmentation

|  | **Week 3** | | **Week 4** | | **Week 5** | |  | |
| --- | --- | --- | --- | --- | --- | --- | --- | --- |
| **Mouse** | SUV_mean_  liver | 40%SUV_max_ (tumor) | SUV_mean_ liver | 40%SUV_max_ (tumor) | SUV_mean_ liver | 40%SUV_max_ (tumor) |  | |
| M1 | 0.46 | 1.60 |  |  |  |  |  | |
| M2 | 0.52 | 1.64 | 0.64 | 1.92 |  |  |  | |
| M3 | 0.46 | 1.44 |  |  | 0.50 | 1.20 |  | |
| M4 |  |  |  |  |  |  |  | |
| M5 |  |  | 0.59 | 1.68 |  |  |  | |
| M6 | 0.49 | 2.08 | 0.58 | 1.60 | 0.47 | 1.48 |  | |
| M7 | 0.59 | 1.84 | 0.48 | 1.56 | 0.38 | 1.40 |  | |
| M8 | 0.55 | 1.44 | 0.51 | 1.52 |  |  |  | |
| M9 | 0.43 | 1.84 | 0.69 | 1.40 |  |  |  | |
| M10 | 0.50 | 1.48 | 0.63 | 1.76 |  |  |  | |
| M11 | 0.48 | 1.52 |  |  |  |  |  | |
| M12 | 0.57 | 1.44 |  |  |  |  |  | |
| M13 | 0.55 | 0.68 |  |  |  |  |  | |
| M14 | 0.52 | 1.44 |  |  |  |  |  | |
| M15 |  |  | 0.48 | 2.00 |  |  |  | |
| M16 |  |  | 0.52 | 1.24 |  |  |  | |
| M17 |  |  | 0.54 | 1.76 |  |  |  | |
| M18 |  |  |  |  | 0.60 | 1.76 | SUV_mean_  liver | 40%  SUV_max_ (tumor) |
| Weeklymean | 0.51 | 1.54 | 0.57 | 1.64 | 0.49 | 1.46 |  |  |
| Overall mean  SD |  |  |  |  |  |  | 0.53  0.06 | 1.57  0.3 |

Abbreviations: SD=standard deviation, SUV=standardized uptake value
