# Supplementary material for: Feasibility and utility of MRI and dynamic 18F-FDG-PET in an orthotopic organoid-based patient-derived mouse model of endometrial cancer
Source: J Transl Med. 2021 Sep 26;19:406. doi: 10.1186/s12967-021-03086-9 (PMC8474962; doi:10.1186/s12967-021-03086-9)
Supplement: Supplementary file 3 — Additional file 3. Calculated mean metabolic tumor volumes (MTV) using the two alternative segmentation algorithms and corresponding mean anatomic tumor volumes from MRI (vMRI). [file 12967_2021_3086_MOESM3_ESM.docx]

**Additional file 3, title:** Calculated mean metabolic tumor volumes (MTV) using the two alternative segmentation algorithms and corresponding mean anatomic tumor volumes from MRI (vMRI).

| PET tumor segmentation (26 scans) | | MRI tumor segmentations (26 scans) |
| --- | --- | --- |
| MTV (mm^3^)  2.5 SUV; fixed threshold | MTV (mm^3^)  40%SUV_max_ | vMRI (mm^3^) |
| 99.2 | 389.1 | 475.2 |

Abbreviations; MTV=metabolic tumor volume, SUV=standardized uptake value, vMRI=tumor volume from MRI
